# Supplementary material for: The Multienzyme Complex Nature of Dehydroepiandrosterone Sulfate Biosynthesis
Source: Int J Mol Sci. 2024 Feb 8;25(4):2072. doi: 10.3390/ijms25042072 (PMC10889563; doi:10.3390/ijms25042072)
Supplement: Supplementary file 1 [file ijms-25-02072-s001.zip › ijms-2795556-supplementary.pdf]

Multienzyme complex nature of dehydroepiandrosterone sulfate biosynthesis

Tumilovich A. M., Yablokov E. O., Mezentsev Y. V.*, Ershov P. V, Basina V. P., Gnedenko O. V., Kaluzhskiy L. A., Tsybruk T. V., Grabovec I. P., Kisel M. S., Shabunya P. S., Soloveva N. A., Vavilov N. E., Gilep A. A., Ivanov A. S.

Correspondence to: Mezentsev Y. V. yu.mezentsev@gmail.com

Table S1. The list of articles used for text mining on any evidence of interactions (functional, co-expression, semantic, etc.) between cytochromes P450 and cytosolic sulfotransferases.

PMID	Title of article	Year of publication
33024080	Alteration of the steroidogenesis in boys with autism spectrum disorders	2020
28752602	Alterations in the steroid biosynthetic pathways in the human prefrontal cortex in mood disorders: A post-mortem study	2018
30326245	Local estrogen metabolism (intracrinology) in endometrial cancer: A systematic review	2019
26940356	Combined steroidogenic characters of fetal adrenal and Leydig cells in childhood adrenocortical carcinoma	2016
20379884	Examination of testicular gene expression patterns in Yorkshire pigs with high and low levels of boar taint	2010
32590918	Expression of Key Androgen-Activating Enzymes in Ovarian Steroid Cell Tumor, Not Otherwise Specified	2020
23197595	Steroidogenic enzyme expression in the human fetal liver and potential role in the endocrinology of pregnancy	2013
29248761	Low-dose metformin exposure causes changes in expression of endocrine disruption-associated genes	2018
28690541	The Significance of the Sulfatase Pathway for Local Estrogen Formation in Endometrial Cancer	2017
19551860	Common germline polymorphisms in COMT, CYP19A1, ESR1, PGR, SULT1E1 and STS and survival after a diagnosis of breast cancer	2009
26704533	Preliminary evidence of altered steroidogenesis in women with Alzheimer's disease: Have the patients "OLDER" adrenal zona reticularis?	2016
19776291	Association of testicular germ cell tumor with polymorphisms in estrogen receptor and steroid metabolism genes	2010
32346900	Interleukin-6 increases adrenal androgen release by regulating the expression of steroidogenic proteins in NCI-H295R cells	2020
21251947	Disrupting effects of bifenthrin on ovulatory gene expression and prostaglandin synthesis in rat ovarian granulosa cells	2011
30981973	Temporal expression pattern of steroid-metabolizing enzymes in bovine COC during in vitro maturation employing different gonadotropin concentrations	2019
36296565	Resveratrol Analogues as Selective Estrogen Signaling Pathway Modulators: Structure-Activity Relationship	2020
25138635	Corpora lutea of pregnant and pseudopregnant domestic cats reveal similar steroidogenic capacities during the luteal life span	2014
29196065	Steroidogenic enzymes, their products and sex steroid receptors during testis development and spermatogenesis in the domestic cat (Felis catus)	2018
31849283	Germline variants and response to systemic therapy in advanced prostate cancer	2020
29588428	SULFATION PATHWAYS: Expression of SULT2A1, SULT2B1 and HSD3B1 in the porcine testis and epididymis	2018

28866654	Hydroxylation and sulfation of sex steroid hormones in inflammatory liver	2017
33815270	Androgen and Luteinizing Hormone Stimulate the Function of Rat Immature Leydig Cells Through Different Transcription Signals	2021
26194504	Is idiopathic hirsutism (IH) really idiopathic? mRNA expressions of skin steroidogenic enzymes in women with IH	2015
27262986	Impact of high-fat diet on liver genes expression profiles in mice model of nonalcoholic fatty liver disease	2016
30217785	The sulfatase pathway as estrogen supply in endometrial cancer	2018
37468957	Abnormal DNA methylation within genes of the steroidogenesis pathway two years after paediatric critical illness and association with stunted growth in height further in time	2023
33421752	Evaluation of testicular toxicity upon fetal exposure to bisphenol A using an organ culture method	2021
20100319	Transcript profiling of candidate genes in testis of pigs exhibiting large differences in androstenone levels	2010
30590593	Characterization of Human Adrenal Steroidogenesis During Fetal Development	2019
27940297	Increased levels of enzymes involved in local estradiol synthesis in chronic obstructive pulmonary disease	2017
36279747	Unhatched bovine blastocysts express all transcripts of the estrogen biosynthetic pathway, but steroid hormone synthesis could not yet be demonstrated	2023
33915228	Targeted RNA sequencing of adrenal zones using immunohistochemistry-guided capture of formalin-fixed paraffin-embedded tissue	2021
37440461	An integrated single-cell analysis of human adrenal cortex development	2023
24576611	Development of adrenal cortical zonation and expression of key elements of adrenal androgen production in the chimpanzee (Pan troglodytes) from birth to adulthood	2014
18318428	CYP1A1, SULT1A1, and SULT1E1 polymorphisms are risk factors for endometrial cancer susceptibility	2008
27854074	Resveratrol and its methoxy derivatives modulate the expression of estrogen metabolism enzymes in breast epithelial cells by AhR down-regulation	2017
17080404	Steroidogenic gene expression in H295R cells and the human adrenal gland: adrenotoxic effects of lindane in vitro	2006
18505908	Analysis of mRNA expression for steroidogenic enzymes in the remaining adrenal cortices attached to adrenocortical adenomas	2008
22542949	Androgen metabolism and JAK/STAT pathway genes and prostate cancer risk	2012
21533175	Eight common genetic variants associated with serum DHEAS levels suggest a key role in ageing mechanisms	2011
27085553	Expression of steroidogenic enzymes and their transcription factors in cortisol-producing adrenocortical adenomas: immunohistochemical analysis and quantitative real-time polymerase chain reaction studies	2016
19443782	MAPK3/1 (ERK1/2) in ovarian granulosa cells are essential for female fertility	2009

22427816	Knockdown of SF-1 and RNF31 Affects Components of Steroidogenesis, TGFβ, and Wnt/β-catenin Signaling in Adrenocortical Carcinoma Cells	2012
30995907	Androgen receptor expression is required to ensure development of adult Leydig cells and to prevent development of steroidogenic cells with adrenal characteristics in the mouse testis	2019
23913208	Survival prediction based on inherited gene variation analysis	2014
33024080	Alteration of the steroidogenesis in boys with autism spectrum disorders	2020
24031161	Comprehensive Assessment and Network Analysis of the Emerging Genetic Susceptibility Landscape of Prostate Cancer	2013
27623070	Age-dependent Increases in Adrenal Cytochrome b5 and Serum 5-Androstenediol-3-sulfate	2016
23709748	Low Systemic Testosterone Levels Induce Androgen Maintenance in Benign Rat Prostate Tissue	2014
25543021	Regulation of androgen biosynthesis - A short review and preliminary results from the hyperandrogenic starvation NCI-H295R cell model	2015
22271526	Adrenal androgen production in catarrhine primates and the evolution of adrenarche	2012
29392307	Testosterone Pathway Genetic Polymorphisms in Relation to Primary Open-Angle Glaucoma: An Analysis in Two Large Datasets	2018
24662082	Rooibos flavonoids inhibit the activity of key adrenal steroidogenic enzymes, modulating steroid hormone levels in H295R cells	2014
28459107	A genomic atlas of human adrenal and gonad development	2017
24616772	Benign adrenal adenomas secreting excess mineralocorticoids and glucocorticoids	2013
29228596	CYP19A1 polymorphisms associated with coronary artery disease and circulating sex hormone levels in a Chinese population	2017
23123734	Estrogen-related receptor α in normal adrenal cortex and adrenocortical tumors: involvement in development and oncogenesis	2013
25404712	Autocrine androgen action is essential for Leydig cell maturation and function, and protects against late-onset Leydig cell apoptosis in both mice and men	2014
34304130	Yangonin modulates lipid homeostasis, ameliorates cholestasis and cellular senescence in alcoholic liver disease via activating nuclear receptor FXR	2021
28954628	Transcriptome analysis reveals differences in mechanisms regulating cessation of luteal function in pregnant and non-pregnant dogs	2017
32428001	Sex-biased genetic programs in liver metabolism and liver fibrosis are controlled by EZH1 and EZH2	2020
24223173	Identification of estrogen target genes during zebrafish embryonic development through transcriptomic analysis	2013
20833731	Ex3αERKO Male Infertility Phenotype Recapitulates the αERKO Male Phenotype	2010
26865584	Adrenal-derived 11-Oxygenated 19-Carbon Steroids are the Dominant Androgens in Classic 21-Hydroxylase Deficiency	2016

24281767	Hypospadias and variants in genes related to sex hormone biosynthesis and metabolism	2015
31357645	Activation of Adrenal Steroidogenesis and an Improvement of Mood Balance in Postmenopausal Females after Spa Treatment Based on Physical Activity	2019
37125608	Primary Aldosteronism: Spatial Multiomics Mapping of Genotype-Dependent Heterogeneity and Tumor Expansion of Aldosterone-Producing Adenomas	2023
28939879	Differential expression and co-expression gene networks reveal candidate biomarkers of boar taint in non-castrated pigs	2017
18497089	Gene expression of 17beta-estradiol-metabolizing isozymes: comparison of normal human mammary gland to normal human liver and to cultured human breast adenocarcinoma cells	2008
24011194	Transcriptomic analysis of the porcine endometrium during early pregnancy and the estrous cycle	2013
21146967	The suitability of rat hepatoma cell line H4IIE for evaluating the potentials of compounds to induce CYP3A23 expression	2012
20887769	Disturbed expression of phase I and phase II estrogen-metabolizing enzymes in endometrial cancer: lower levels of CYP1B1 and increased expression of S-COMT	2011
16985250	Estrogen sulfation genes, hormone replacement therapy, and endometrial cancer risk	2006
17945481	Regulation of the adrenal androgen biosynthesis	2007
21828262	Regulation of estrogen sulfotransferase expression by confluence of MCF10A breast epithelial cells: role of the aryl hydrocarbon receptor	2011
23277161	Disturbed balance between phase I and II metabolizing enzymes in ovarian endometriosis: a source of excessive hydroxy-estrogens and ROS?	2013
19497978	The mediator complex subunit 1 enhances transcription of genes needed for adrenal androgen production	2009
15681896	Effects of NO-1886 (Ibrolipim), a lipoprotein lipase-promoting agent, on gene induction of cytochrome P450s, carboxylesterases, and sulfotransferases in primary cultures of human hepatocytes	2004
23685396	11β-hydroxyandrostenedione, the product of androstenedione metabolism in the adrenal, is metabolized in LNCaP cells by 5α-reductase yielding 11β-hydroxy-5α-androstanedione	2013
17921479	Estrogen metabolizing enzymes in endometrium and endometriosis	2007
23470964	Molecular pathways: Inhibiting steroid biosynthesis in prostate cancer	2013
16755106	New development in intracrinology of breast carcinoma	2006
28368480	Genetic and Histopathologic Intertumor Heterogeneity in Primary Aldosteronism	2017
19130396	Expression of enzymes involved in synthesis and metabolism of estradiol in human breast as studied by immunocytochemistry and in situ hybridization	2009
14623518	Breast cancer tissue estrogens and their manipulation with aromatase inhibitors and inactivators	2003
18690485	Differential regulation of dehydroepiandrosterone and estrogen on bone and uterus in ovariectomized mice	2009

21740958	The role of estrogen-metabolizing enzymes and estrogen receptors in human epidermis	2011
17548088	In vitro effect of dehydroepiandrosterone sulfate on steroid receptors, aromatase, cyclooxygenase-2 expression, and steroid hormone production in preovulatory human granulosa cells	2007
23848148	Changes in levels of gene expression in human aortal intima during atherogenesis	2013
34107159	Effects of Lemborexant on the Pharmacokinetics of Oral Contraceptives: Results From a Phase 1 Drug-Drug Interaction Study in Healthy Females	2021
31536780	Evaluating the effects on steroidogenesis of estragole and trans-anethole in a feto-placental co-culture model	2019
18725155	CYP11A1 and CYP17 promoter polymorphisms associate with hyperandrogenemia in polycystic ovary syndrome	2009
22369716	Breast cancer therapy based on melatonin	2012
15748827	Effect of nomegestrol acetate on estrogen biosynthesis and transformation in MCF-7 and T47-D breast cancer cells	2005
35713964	Ziritaxestat Drug-Drug Interaction with Oral Contraceptives: Role of SULT1E1 Inhibition	2022
16530336	Androgen excess in women--a health hazard?	2006
11306177	Temporal and spatial distribution of corticosteroidogenic enzymes immunoreactivity in developing human adrenal	2001
12563679	Serum levels of pregnenolone and 17-hydroxypregnenolone in patients with rheumatoid arthritis and systemic lupus erythematosus: relation to other adrenal hormones	2003
18663016	Excess estrogen sulfoconjugation as the possible cause for a poor sign of parturition in pregnant cows carrying somatic cell clone fetuses	2008
36413950	Reconstitution of human adrenocortical specification and steroidogenesis using induced pluripotent stem cells	2022
25336526	Wt1 dictates the fate of fetal and adult Leydig cells during development in the mouse testis	2014
20505544	Effect of hormone metabolism genotypes on steroid hormone levels and menopausal symptoms in a prospective population-based cohort of women experiencing the menopausal transition	2010
16303840	Estrogen-metabolizing enzymes in breast cancers from women over the age of 80 years	2006
16293666	Dehydroepiandrosterone sulfate is neuroprotective when administered either before or after injury in a focal cortical cold lesion model	2006
34944390	Altered Steroidome in Women with Gestational Diabetes Mellitus: Focus on Neuroactive and Immunomodulatory Steroids from the 24th Week of Pregnancy to Labor	2021
18589890	The prevalence of 21-hydroxylase deficiency in adrenal incidentalomas - hormonal and mutation screening	2008
12959982	GATA-6 is expressed in the human adrenal and regulates transcription of genes required for adrenal androgen biosynthesis	2003
23900415	Protein kinase C-induced activin A switches adrenocortical steroidogenesis to aldosterone by suppressing CYP17A1 expression	2013

19085698	The frequency of CYP 21 gene mutations in Turkish women with hyperandrogenism	2009
18505907	The synergistic effect of sex hormone-binding globulin and aromatase genes on polycystic ovary syndrome phenotype	2008
19308726	Expression of estrogenicity genes in a lineage cell culture model of human breast cancer progression	2010
33441255	Estrogens and development of the rete testis, efferent ductules, epididymis and vas deferens	2022
25817828	Local estrogen metabolism in epithelial ovarian cancer suggests novel targets for therapy	2015
10067824	Regulation of estrogen sulfotransferase expression in Leydig cells by cyclic adenosine 3',5'-monophosphate and androgen	1999
14523365	Evaluation of the pituitary-adrenal axis in hyperandrogenic women with polycystic ovary syndrome	2003
15705377	Prevalence of CYP21 mutations and IRS1 variant among women with polycystic ovary syndrome and adrenal androgen excess	2005
36605939	Role of aerobic exercise in ameliorating NASH: Insights into the hepatic thyroid hormone signaling and circulating thyroid hormones	2022
34094659	Gene polymorphism-related differences in the outcomes of abiraterone for prostate cancer: a systematic overview	2021
18774939	Comparative contents of mRNAs of sex steroid receptors and enzymes of their metabolism in arterial walls of men	2008
29684161	Impact of heat stress during the follicular phase on porcine ovarian steroidogenic and phosphatidylinositol-3 signaling	2018
19962254	Breast cancer and steroid metabolizing enzymes: the role of progestogens	2009
35059953	Estrogen Sulfotransferase is Highly Expressed in Vascular Endothelial Cells Overlying Atherosclerotic Plaques	2022
37484942	Dexamethasone affects human fetal adrenal steroidogenesis and subsequent ACTH response in an ex vivo culture model	2023
29429410	The human fetal adrenal produces cortisol but no detectable aldosterone throughout the second trimester	2018
18089598	Expression of aromatase and estrogen sulfotransferase in eutopic and ectopic endometrium: evidence for unbalanced estradiol production in endometriosis	2007
17661084	Expression of aromatase and estrogen sulfotransferase in preinvasive and invasive breast cancer	2008
35442744	The developmental origin and the specification of the adrenal cortex in humans and cynomolgus monkeys	2022
11155097	Developmental changes in steroidogenic enzymes in human postnatal adrenal cortex: immunohistochemical studies	2000
20178799	Mice lacking Mrp1 have reduced testicular steroid hormone levels and alterations in steroid biosynthetic enzymes	2010
36976310	Effect of Dexamethasone on Abiraterone Pharmacokinetics in Mice: Determined by LC/MS Analysis	2023
25875613	Human induced hepatic lineage-oriented stem cells: autonomous specification of human iPS cells toward hepatocyte-like cells without any exogenous differentiation factors	2015

19250194	New developments in intracrinology of human breast cancer: estrogen sulfatase and sulfotransferase	2009
35982918	An approach to uncover the relationship between 17b-estradiol and ESR1/ESR2 ratio in the regulation of canine corpus luteum	2022
27809697	Determination of 17OHPreg and DHEAS by LC-MS/MS: Impact of Age, Sex, Pubertal Stage, and BMI on the Δ5 Steroid Pathway	2017
27531568	Role of steroid sulfatase in steroid homeostasis and characterization of the sulfated steroid pathway: Evidence from steroid sulfatase deficiency	2016
9539798	Dehydroepiandrosterone: a potential signalling molecule for neocortical organization during development	1998
15355916	Steroid sulfatase and estrogen sulfotransferase in human endometrial carcinoma	2004
33911053	Suppressed estrogen supply via extra-ovarian progesterone receptor membrane component 1 in menopause	2021
33064293	Impact of Human SULT1E1 Polymorphisms on the Sulfation of 17β-Estradiol, 4-Hydroxytamoxifen and Diethylstilbestrol by SULT1E1 Allozymes	2022
18467089	In situ estrogen metabolism in proliferative endometria from untreated women with polycystic ovarian syndrome with and without endometrial hyperplasia	2008
36473578	Novel insights into bile acid detoxification via CYP, UGT and SULT enzymes	2023
15878968	Transcriptional regulation of dehydroepiandrosterone sulfotransferase (SULT2A1) by estrogen-related receptor alpha	2005
34926449	Arbutin Alleviates the Liver Injury of α-Naphthylisothiocyanate-induced Cholestasis Through Farnesoid X Receptor Activation	2021
15482786	Role of a CYP17 polymorphism in the regulation of circulating dehydroepiandrosterone sulfate levels in women with polycystic ovary syndrome	2004
26047979	Preliminary study of FMO1, FMO5, CYP21, ESR1, PLIN2 and SULT2A1 as candidate gene for compounds related to boar taint	2015
17466517	Steroid pathway and oestrone sulphate production in canine inflammatory mammary carcinoma	2007
31169883	Genetic Association Study of Eight Steroid Hormones and Implications for Sexual Dimorphism of Coronary Artery Disease	2019
21084398	Clinical, biochemical, and molecular characterization of macronodular adrenocortical hyperplasia of the zona reticularis: a new syndrome	2011
31425786	Essential oils disrupt steroidogenesis in a feto-placental co-culture model	2019
25767055	Dienogest reduces HSD17β1 expression and activity in endometriosis	2015
19141651	Steroid sulfatase and estrogen sulfotransferase in colon carcinoma: regulators of intratumoral estrogen concentrations and potent prognostic factors	2009
23476785	The sulfatase pathway for estrogen formation: targets for the treatment and diagnosis of hormone-associated tumors	2013

33748934	Pharmacokinetic and Pharmacodynamic Profiles of Ethinylestradiol/Norgestimate Combination or Norethindrone upon Coadministration with Elagolix 150 mg Once Daily in Healthy Premenopausal Women	2021
16014403	Corticotropin-releasing hormone (CRH) and urocortin act through type 1 CRH receptors to stimulate dehydroepiandrosterone sulfate production in human fetal adrenal cells	2005
26888303	n-butylparaben induces male reproductive disorders via regulation of estradiol and estrogen receptors	2016
19567527	Adrenal function during childhood and puberty in daughters of women with polycystic ovary syndrome	2009
37110661	Hepatotoxic Components Effect of Chebulae Fructus and Associated Molecular Mechanism by Integrated Transcriptome and Molecular Docking	2023
16168635	Treatment with high-dose estrogen (diethylstilbestrol) significantly decreases plasma estrogen and androgen levels but does not influence in vivo aromatization in postmenopausal breast cancer patients	2005
37949197	Diuron-induced fetal Leydig cell dysfunction in in vitro organ cultured fetal testes	2023
9870265	C19 steroids estrogenic activity in human breast cancer cell lines: importance of dehydroepiandrosterone sulfate at physiological plasma concentration	1998
33340681	Steroid metabolome profiling of follicular fluid in normo- and hyperandrogenic women with polycystic ovary syndrome	2021
37268990	Dehydroepiandrosterone supplementation and the impact of follicular fluid metabolome and cytokinome profiles in poor ovarian responders	2023
34107842	SULT genetic polymorphisms: physiological, pharmacological and clinical implications	2021
29467139	SULFATION PATHWAYS: Formation and hydrolysis of sulfonated estrogens in the porcine testis and epididymis	2018
17408424	Benign cortisol-secreting adrenocortical adenomas produce small amounts of androgens	2007
19022235	Expression of 17beta-hydroxysteroid dehydrogenases and other estrogen-metabolizing enzymes in different cancer cell lines	2009
37110661	Hepatotoxic Components Effect of Chebulae Fructus and Associated Molecular Mechanism by Integrated Transcriptome and Molecular Docking	2023
37953901	In Situ Spatial Reconstruction of Distinct Normal and Pathological Cell Populations Within the Human Adrenal Gland	2023
33753083	An in vitro investigation of endocrine disrupting potentials of ten bisphenol analogues	2021
33002589	Intratumoral heterogeneity of the tumor cells based on in situ cortisol excess in cortisol-producing adenomas; ~An association among morphometry, genotype and cellular senescence~	2020
27923582	High mRNA levels of 17β-hydroxysteroid dehydrogenase type 1 correlate with poor prognosis in endometrial cancer	2017
22422661	Acute effect of high-dose isoflavones from Pueraria lobata (Willd.) Ohwi on lipid and bone metabolism in ovariectomized mice	2012

37881184	The zhuyu pill relieves rat cholestasis by regulating the mRNA expression of lipid and bile metabolism associated genes	2023
33865853	Elucidation of metabolic pathways of 25-hydroxyvitamin D3 mediated by CYP24A1 and CYP3A using Cyp24a1 knockout rats generated by CRISPR/Cas9 system	2021
35806403	Per1/Per2 Disruption Reduces Testosterone Synthesis and Impairs Fertility in Elderly Male Mice	2022

Table S2. A spectrum of potential partner proteins of SULT1E1 isolated from rat liver lysate.

Peptides	Unique peptides	Sequence coverage [%]	Mol. weight [kDa]	Score	Intensity	MS/MS count	Uniprot ID	Peptide sequences	Protein name
1	1	4.1	37.331	33.957	4835200	1	P54313	SELEQLRQEAEQLR	Guanine nucleotide-binding protein G(I)/G(S)/G(T) subunit beta-2<br>Guanine nucleotide-binding protein subunit beta-4
2	2	23.9	15.355	20.545	28254000	2	P00173	AEQSDKDVKYYTLEEIQK<br>TYIIGELHPDDRSK	Cytochrome b5 OS=Rattus norvegicus
1	1	5.5	39.618	13.263	10271000	2	P00884	ISDQCPSSLAIQENANALAR	Fructose-bisphosphate aldolase B
2	2	4.9	46.496	63.334	136930000	4	P09034	TQDPAKAPNTPDVLEIEFK<br>TQDPAKAPNTPDVLEIEFKK	Argininosuccinate synthase
1	1	6.8	23.504	6.4743	2695200	1	P09527	EAINVEQAFQTIAR	Ras-related protein Rab-7a
1	1	2.2	57.076	6.0321	3769800	1	P12939	FGDIAPLNLPR	Cytochrome P450 2D10<br>Cytochrome P450 2D1
4	4	16.2	33.906	40.594	171400000	5	P17988	CPGVPSGLETLEETPAPR<br>EFSRPPLVHVK<br>MEFSRPPLVHVK<br>SLPEETVDSIVHHTSFK	Sulfotransferase 1A1
2	2	8.7	38.201	70.811	35961000	2	P29147	TIQLNVCNSEEVEKAVETVR<br>VVNISSMLGR	D-beta-hydroxybutyrate dehydrogenase. mitochondrial
1	1	1.2	85.338	5.9824	8107600	1	P30835	ATVDLEKLR	ATP-dependent 6-phosphofructokinase. liver type
1	1	1.9	54.081	6.1273	7729200	1	P30839	MERQVQRLR	Aldehyde dehydrogenase family 3 member A2
4	4	18.8	35.764	107.72	357530000	12	P50237	AGTTWTQEIVDMIQNDGDVQKCQR<br>KMAGSNITFRTEI<br>MAGSNITFRTEI<br>SLEKMKDLHLGEQDLQPETR	Sulfotransferase 1C1

4	4	13.7	34.835	67.922	184560000	6	P52847	GTAEDVFRK<br>GTAEDVFRKDLK<br>IANFLDKTLDEHTLER<br>SGVELLKKTPSPR	Sulfotransferase 1B1
4	4	3.2	101.44	47.038	70458000	4	Q64380	AAVARGAQVIENCAVTGIR<br>GAQVIENCAVTGIR<br>GAQVIENCAVTGIRVR<br>VRTDDFGVRR	Sarcosine dehydrogenase. mitochondrial
2	2	3.7	34.859	254.32	430230000	3	Q9WUW9	ALAPELSR<br>ALAPELSRQTK	Sulfotransferase 1C2A<br>Sulfotransferase 1C2

Table S3. A list of amino acid residues (a.a.r.) in the protein interfaces of SULT1E1 complexes with CYP17A1 and CYB5A (models).

dimeric SULT1E1/CYP17A1			
SULT1E1(chain A):CYP17A1 (interface1)		SULT1E1(chain B):CYP17A1 (interface2)	
SULT1E1(chain A)	CYP17A1	SULT1E1(chain B)	CYP17A1
ALA146, LYS232, THR240, THR241, *LEU242*, PRO243, *ASP244*, GLU245, *ASN248*, GLN249, ***LYS250***, LEU251, SER252, PRO253	LYS136, ASP137, GLY138, *ASP139*, *GLN140*, LYS141, **GLU143**, LYS144, THR343, ILE344, SER345, ASN348, ARG349, GLN450	***MET1***, ASN2, SER3, GLU4, ASP6, TYR7, **GLU9**, LYS10, ALA146, ASN150, GLY152, SER153, *GLU156*, LYS160, ASP244, **GLU245**, *ASN248*, *LYS250*, LEU251	PRO62, **HIS78**, HIS79, *GLN80*, LEU81, LYS83, GLU84, LYS88, GLN323, LYS327, ASN402, LYS404, GLU405, **HIS407**, GLN408, PHE412, MET413, ***GLU415***, ARG416, LEU418, *ASN419*, PRO420, ALA421, THR423, GLN424, ILE426, SER427, PRO428, VAL430
dimeric SULT1E1/CYB5A			
SULT1E1(chain A):CYB5A (interface1)		SULT1E1(chainB):CYB5A (interface2)	
SULT1E1(chain A)	CYB5A	SULT1E1(chain B)	CYB5A
TYR139, TYR140, LEU143, *ASN150*, GLY152, SER153, PHE154, ASP244, GLU245, *ASN248*, *LYS250*, LEU251, SER252	HIS31, ASP58, THR60, GLU61, *GLU64*, ASP65, VAL66, GLY67, LEU92, *ASN93*, LYS94, PRO95	MET1, SER3, ASP6, TYR139, LEU143, ALA146, *ASN150*, GLY152, SER153, GLU156, ASN248, LYS250, LEU251	SER23, PHE40, GLU42, GLU43, HIS44, PRO45, GLY46, GLY47, GLU48, *GLU49*, VAL50, ARG52, MET75, ILE103
monomeric SULT1E1/CYP17A1			

SULT1E1	CYP17A1
MET1, SER3, ***GLU4***, ASP6, TYR139, *TYR140*, LEU143, ASN150, GLY152, SER153, PHE154, PRO155, GLU156, LYS232, LYS250, LEU251, SER252, PRO253, ARG256, ***LYS257***, ILE259, LYS264, *ASN265*, HIS266, THR268, VAL269, ALA270, GLU273, LYS274, GLU293	LYS83, ILE87, ***LYS91***, GLN122, ARG125, ARG126, MET129, ALA130, ALA133, LEU134, *LYS136*, ASP137, GLY138, ***ASP139***, ASP257, PRO280, ASP281, ILE344, ARG347, ASN348, ARG358, GLN424, ILE426, SER427, SER429, VAL430, TYR432, LEU433, ALA437, GLY438, PRO439, SER441, GLU445, ILE446, ARG449, GLN450
monomeric SULT1E1/CYB5A	
SULT1E1	CYB5A
LEU143, ASN150, GLY152, SER153, PHE154, PRO155, GLU156, PHE228, GLN229, **LYS232**, GLN249, *LYS250*, *LEU251*, SER252, PRO253, **ARG256**, ***LYS257***, ILE259, LYS264, ASN265, ***HIS266***, PHE267, THR268, *VAL269*, ALA270	GLY46, GLY47, ***GLU49***, VAL50, ARG52, ***GLU53***, GLN54, GLU61, ASN62, *ASP65*, VAL66, PRO95, PRO96, GLU97, THR98, LEU99, ILE100, THR101, *THR102*, ILE103, **ASP104**, SER105, SER107, TRP110, THR111, ILE115

a.a.r., forming salt bridges and H-bonds, are highlighted with bold and italic types, respectively. a.a.r., whose involvement in the formation of hydrogen or electrostatic bonds is typical for most of the models predicted, are highlighted with green color.

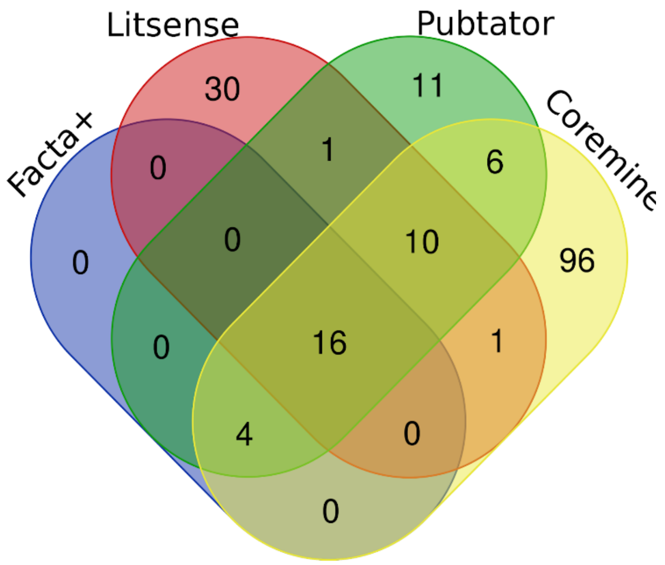

Figure S1. Venn diagrams showing number of articles found using four different text mining web-based tools.

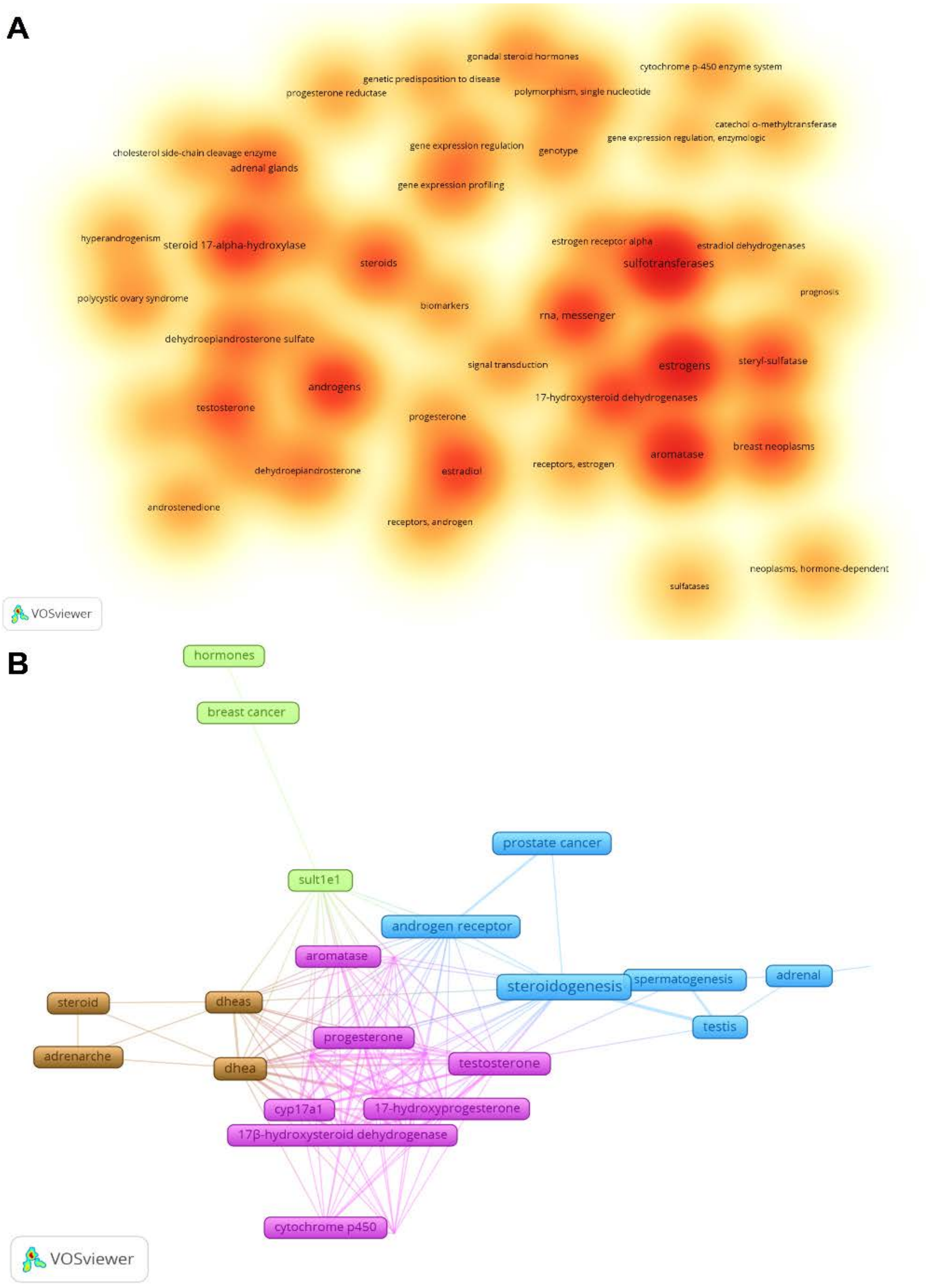

Figure S2. (A) – a density map of keywords associated with SULTs and CYPs in 175 articles from the target sample. (B) – a map of co-occurrence of keywords associated with SULTs and CYPs in 175 articles of the target sample. Designations: cluster 1 (red), cluster 2 (green), cluster 3 (blue), cluster 4 (yellow).

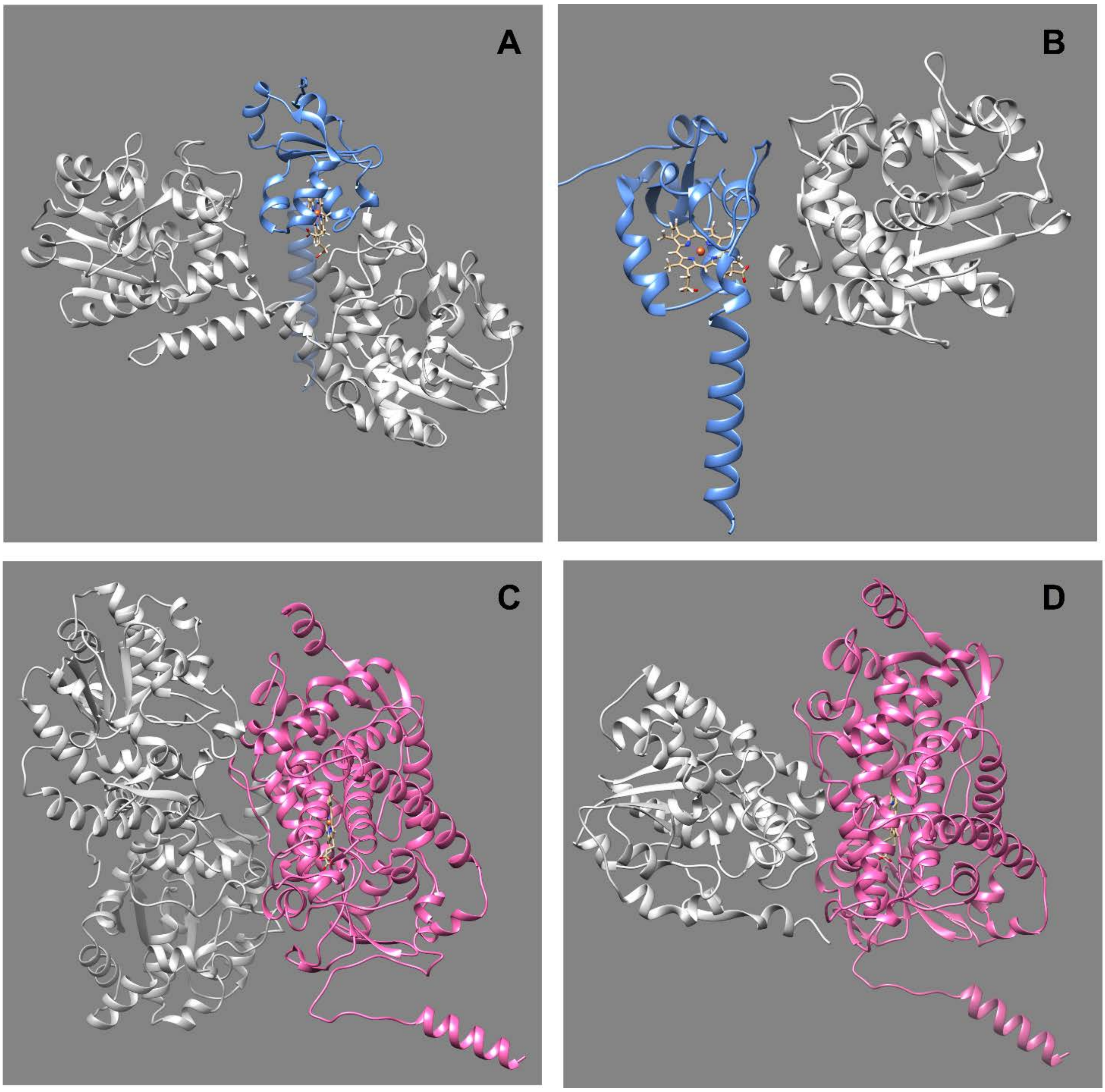

Figure S3. Models of dimeric (A, C) and monomeric (B, D) SULT1E1 with CYB5A (A, B) and CYP17A1 (C, D) predicted in the AlphaFold 2 software.

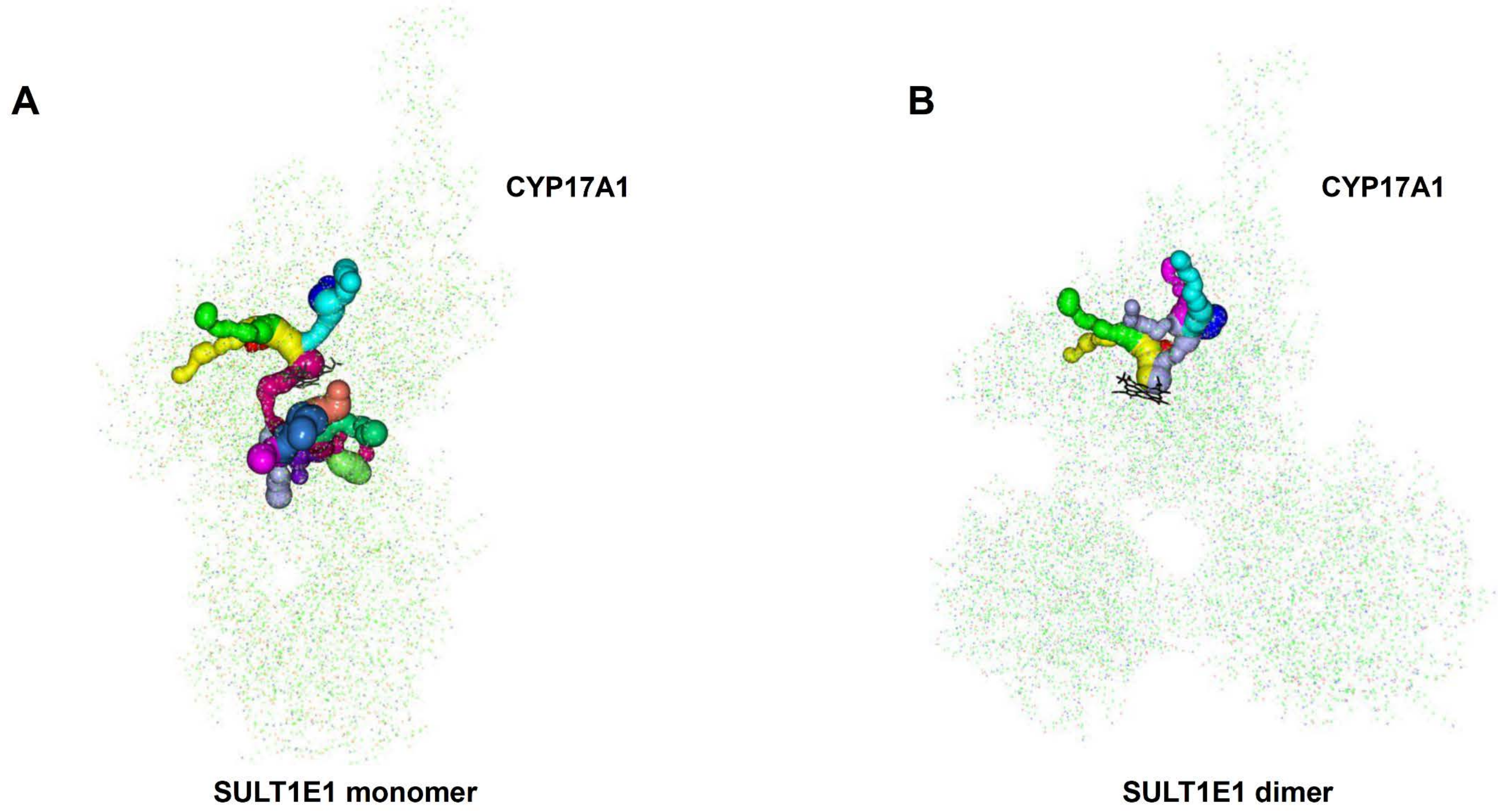

Figure S4. A set of calculated channels in CYP17A1 in complexes with the monomer (A) and dimer (B) forms of SULT1E1. The channels are colored according to the entry point on the surface of CYP17A1. It is possible to overlay different colors on top of each other in case of merging channels together.

A.

```
                          1                                                  50
     CYP17A1       (1)    MWELVALLLLTLAYLFWPKRRCPGAKYPKSLLSLPLVGSLPFLPRHGHMH
  CYP17A1_tr       (1)    ----------------------MAKKTPKSLLSLPLVGSLPFLPRHGHMH
                          51                                                100
     CYP17A1      (51)    NNFFKLQKKYGPIYSVRMGTKTTVIVGHHQLAKEVLIKKGKDFSGRPQMA
  CYP17A1_tr      (29)    NNFFKLQKKYGPIYSVRMGTKTTVIVGHHQLAKEVLIKKGKDFSGRPQMA
                          101                                               150
     CYP17A1     (101)    TLDIASNNRKGIAFADSGAHWQLHRRLAMATFALFKDGDQKLEKIICQEI
  CYP17A1_tr      (79)    TLDIASNNRKGIAFADSGAHWQLHRRLAMATFALFKDGDQKLEKIICQEI
                          151                                               200
     CYP17A1     (151)    STLCDMLATHNGQSIDISFPVFVAVTNVISLICFNTSYKNGDPELNVIQN
  CYP17A1_tr     (129)    STLCDMLATHNGQSIDISFPVFVAVTNVISLICFNTSYKNGDPELNVIQN
                          201                                               250
     CYP17A1     (201)    YNEGIIDNLSKDSLVDLVPWLKIFPNKTLEKLKSHVKIRNDLLNKILENY
  CYP17A1_tr     (179)    YNEGIIDNLSKDSLVDLVPWLKIFPNKTLEKLKSHVKIRNDLLNKILENY
                          251                                               300
     CYP17A1     (251)    KEKFRSDSITNMLDTLMQAKMNSDNGNAGPDQDSELLSDNHILTTIGDIF
  CYP17A1_tr     (229)    KEKFRSDSITNMLDTLMQAKMNSDNGNAGPDQDSELLSDNHILTTIGDIF
                          301                                               350
     CYP17A1     (301)    GAGVETTTSVVKWTLAFLLHNPQVKKKLYEEIDQNVGFSRTPTISDRNRL
  CYP17A1_tr     (279)    GAGVETTTSVVKWTLAFLLHNPQVKKKLYEEIDQNVGFSRTPTISDRNRL
                          351                                               400
     CYP17A1     (351)    LLLEATIREVLRLRPVAPMLIPHKANVDSSIGEFAVDKGTEVIINLWALH
  CYP17A1_tr     (329)    LLLEATIREVLRLRPVAPMLIPHKANVDSSIGEFAVDKGTEVIINLWALH
                          401                                               450
     CYP17A1     (401)    HNEKEWHQPDQFMPERFLNPAGTQLISPSVSYLPFGAGPRSCIGEILARQ
  CYP17A1_tr     (379)    HNEKEWHQPDQFMPERFLNPAGTQLISPSVSYLPFGAGPRSCIGEILARQ
                          451                                               500
     CYP17A1     (451)    ELFLIMAWLLQRFDLEVPDDGQLPSLEGIPKVVFLIDSFKVKIKVRQAWR
  CYP17A1_tr     (429)    ELFLIMAWLLQRFDLEVPDDGQLPSLEGIPKVVFLIDSFKVKIKVRQAWR
                          501          514
     CYP17A1     (501)    EAQAEGSTHHHHHH
  CYP17A1_tr     (479)    EAQAEGSTHHHHHH
```

B.

```
                          1                                                  50
     SULT1E1       (1)    HHHHHHMNSELDYYEKFEEVHGILMYKDFVKYWDNVEAFQARPDDLVIAT
     SULT2A1       (1)    ---------MSDDFLWFEGIAFPTMGFRSETLRKVRDEFVIRDEDVIILT
                          51                                                100
     SULT1E1      (51)    YPKSGTTWVSEIVYMIYKEGDVEKCKEDVIFNRIPFLECRKENLMNGVKQ
     SULT2A1      (42)    YPKSGTNWLAEILCLMHSKGDAKWIQSVPIWERSPWVES-----EIGYTA
                          101                                               150
     SULT1E1     (101)    LDEMNSPRIVKTHLPPELLPASFWEKDCKIIYLCRNAKDVAVSFYYFFLM
     SULT2A1      (87)    LSETESPRLFSSHLPIQLFPKSFFSSKAKVIYLMRNPRDVLVSGYFFWKN
                          151                                               200
     SULT1E1     (151)    VAGHPNPGSFPEFVEKFMQGQVPYGSWYKHVKSWWEKGKSPRVLFLFYED
     SULT2A1     (137)    MKFIKKPKSWEEYFEWFCQGTVLYGSWFDHIHGWMPMREEKNFLLLSYEE
                          201                                               250
     SULT1E1     (201)    LKEDIRKEVIKLIHFLERKPSEELVDRIIHHTSFQEMKNNPSTNYTTLPD
     SULT2A1     (187)    LKQDTGRTIEKICQFLGRTLEPEELNLILKNSSFQSMKENKMSNYSLLSV
                          251                                               300
     SULT1E1     (251)    EIMNQKLSPFMRKGITGDWKNHFTVALNEKFDKHYEQQMKESTLKFRTEI
     SULT2A1     (237)    DYVVDKAQ-LLRKGVSGDWKNHFTVAQAEDFDKLFQEKMADLPRELFPWE
                          301
     SULT1E1     (301)    ------
     SULT2A1     (286)    HHHHHH
```

Figure S5. Comparison of amino acid sequences of the studied proteins using the Align software package (Invitrogen, USA). A. CYP17A1/ CYP17A1tr; B. SULT1E1/ SULT2A1.

- the missing part of the trunked form of CYP17A1.

- the homologous part of the amino acid sequence.

- the identical part of the amino acid sequence.

- artificially added 6xHis-tag, for subsequent purification of proteins by metal-affinity chromatography (Ni-NTA sorbent).

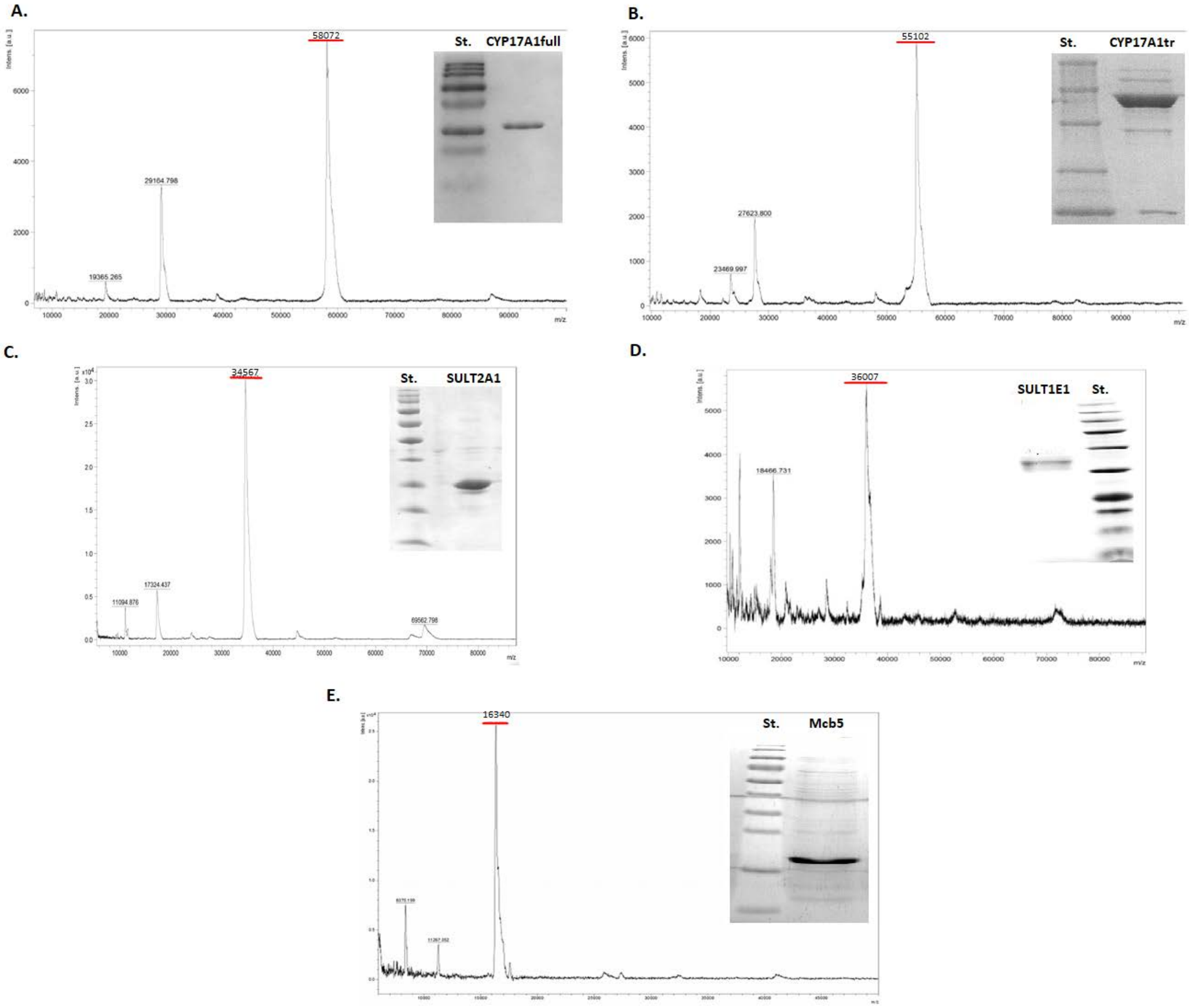
A.
58072
St.
CYP17A1full
29164.798
19365.265
Intens. [a.u.]
m/z
B.
55102
St.
CYP17A1tr
27623.800
23469.997
Intens. [a.u.]
m/z
C.
34567
St.
SULT2A1
17324.437
11094.876
69562.798
Intens. [a.u.]
m/z
D.
36007
SULT1E1
St.
18466.731
Intens. [a.u.]
m/z
E.
16340
St.
Mcb5

Figure S6. Protein purification data. A. Preparation of CYP17A1 full-length with a mass of 58.072 kDa (according to MALDI-TOF mass spectrometry). SDS-PAGE analysis of CYP17A1 full-length protein. St. - protein molecular weight standard (250-10 kDa, Thermo Scientific #26619). B. Preparation of CYP17A1tr with a mass of 55.102 kDa (according to MALDI-TOF mass spectrometry). SDS-PAGE analysis of CYP17A1 tr. protein. St. - protein molecular weight standard (250-10 kDa, Thermo Scientific #26619). C. Preparation of SULT2A1 with a mass of 34.567 kDa (according to MALDI-TOF mass spectrometry). SDS-PAGE analysis of SULT2A1 protein. St. - protein molecular weight standard (250-10kDa, NEB #P7719S). D. Preparation of SULT1E1 with a mass of 36.007 kDa (according to MALDI-TOF mass spectrometry). SDS-PAGE analysis of SULT1E1 protein. St. - protein molecular weight standard (245-11kDa, NEB #P7712S). E. Preparation of Mcb5 with a mass of 16.340 kDa (according to MALDI-TOF mass spectrometry). SDS-PAGE analysis of Mcb5 protein. St. - protein molecular weight standard (180-10kDa, Thermo Scientific #26616).
